# The microbiome and rise of early-onset cancers: knowledge gaps and research opportunities

**DOI:** 10.1080/19490976.2023.2269623

**Published:** 2023-10-30

**Authors:** Kosuke Mima, Tsuyoshi Hamada, Kentaro Inamura, Hideo Baba, Tomotaka Ugai, Shuji Ogino

**Affiliations:** a Department of Gastroenterological Surgery, Graduate School of Medical Sciences, Kumamoto University, Kumamoto, Japan; b Department of Gastroenterology, Graduate School of Medicine, The University of Tokyo, Tokyo, Japan; c Department of Hepato-Biliary-Pancreatic Medicine, The Cancer Institute Hospital, Japanese Foundation for Cancer Research, Tokyo, Japan; d Division of Pathology, The Cancer Institute, Japanese Foundation for Cancer Research, Tokyo, Japan; e Department of Pathology, The Cancer Institute Hospital, Japanese Foundation for Cancer Research, Tokyo, Japan; f Program in MPE Molecular Pathological Epidemiology, Department of Pathology, Brigham and Women’s Hospital and Harvard Medical School, Boston, MA, USA; g Department of Epidemiology, Harvard T.H. Chan School of Public Health, Boston, MA, USA; h Cancer Epidemiology Program, Dana-Farber Harvard Cancer Center, Boston, MA, USA; i Broad Institute of MIT and Harvard, Cambridge, MA, USA; j Cancer Immunology Program, Dana-Farber Harvard Cancer Center, Boston, MA, USA

**Keywords:** Big data, bioinformatics, exposome, inflammation, omics, population health science

## Abstract

Accumulating evidence indicates an alarming increase in the incidence of early-onset cancers, which are diagnosed among adults under 50 years of age, in the colorectum, esophagus, extrahepatic bile duct, gallbladder, liver, stomach, pancreas, as well as the bone marrow (multiple myeloma), breast, head and neck, kidney, prostate, thyroid, and uterine corpus (endometrium). While the early-onset cancer studies have encompassed research on the wide variety of organs, this article focuses on research on digestive system cancers. While a minority of early-onset cancers in the digestive system are associated with cancer-predisposing high penetrance germline genetic variants, the majority of those cancers are sporadic and multifactorial. Although potential etiological roles of diets, lifestyle, environment, and the microbiome from early life to adulthood (i.e. in one’s life course) have been hypothesized, exact contribution of each of these factors remains uncertain. Diets, lifestyle patterns, and environmental exposures have been shown to alter the oral and intestinal microbiome. To address the rising trend of early-onset cancers, transdisciplinary research approaches including lifecourse epidemiology and molecular pathological epidemiology frameworks, nutritional and environmental sciences, multi-omics technologies, etc. are needed. We review current evidence and discuss emerging research opportunities, which can improve our understanding of their etiologies and help us design better strategies for prevention and treatment to reduce the cancer burden in populations.

## Introduction

Carcinomas arising in the digestive system are leading causes of cancer death worldwide.^1^ Accumulating evidence indicates that the incidence and mortality of digestive system cancers diagnosed in patients younger than 50 years of age has dramatically increased in many parts for the world.^2–4^ The term “early-onset cancer” is generally used for adulthood cancer diagnosed under 50 years of age. We used this term and definition for consistency throughout this article. We also use the contrasting term “later-onset cancer” for cancer diagnosed in patients aged 50 years or older. However, it should be noted that clinical, pathological, and molecular features of cancer may not sharply change at the age of 50 years. Early-onset digestive system cancers may be associated with familial clustering or cancer-predisposing germline genetic variants. However, the majority of them appear to be sporadic and multifactorial in their origins.^5–8^ Because younger individuals have longer life expectancies compared to older individuals, developments of strategies for early-onset cancer prevention may have a substantial impact on public health and be cost-effective in a long run. However, there is the scarcity of population-based evidence on risk factors for early-onset cancers necessary to design and implement prevention programs.

**Figure 1.**
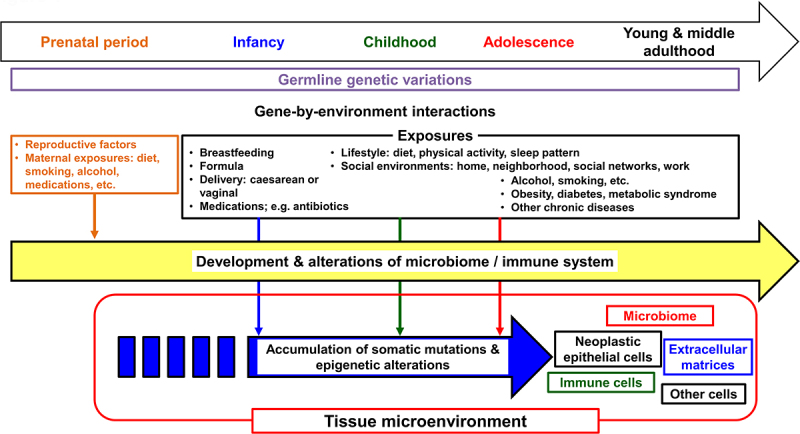
Life course perspective on factors related to early-onset cancers. Early-onset digestive system cancers develop through the accumulation of somatic mutations and epigenetic alterations in tumor cells under interactive influences of germline genetic variations and various exposures, i.e., gene-by-environmental interactions. An interplay between epithelial, microbial, immune, other cells, and extracellular matrices plays a pivotal role in the tumorigenic (and/or anti-tumorigenic) processes. Theoretically, these processes may start from the prenatal period and remain variably operative throughout one’s life course.

Commensal microorganisms in the human body include bacteria, viruses, fungi, and parasites. A tremendous number of bacteria reside in the gastrointestinal tract, especially in the colon and rectum.^9^ The intestinal microbiome has been shown to develop throughout early life and influence the systemic metabolic status and immune system.^10–12^ Maternal exposures, including diet, smoking, alcohol consumption, and medication, as well as infant feeding (breastfeeding vs. formula feeding) have been shown to influence the infant gut microbiome.^13–16^ As digestive system cancers are heterogeneous diseases influenced by exogenous and endogenous factors such as diets, lifestyle, the microbiome, and immune system (Figure 1), an integrative approach is required to elucidate their etiology and pathogenesis.^17–19^ The integration of molecular pathology and epidemiology has generated the transdisciplinary field of molecular pathological epidemiology (MPE),^20^ which aims to link the exposome with specific pathogenic cellular and molecular signatures.^21–23^ The integration of microbiology into the MPE approach can provide a better understanding of the interactions between environment exposures, neoplastic cells, immune cells, and the microbiome during the development and progression of early-onset digestive system cancer.^24,25^ In this review, we summarize current evidence on early-onset digestive system cancers and discuss emerging research opportunities to address the alarming increase in the incidence of early-onset digestive system cancers worldwide.

## Current evidence of early-onset digestive system cancers

### Esophageal cancer

There are two main histological subtypes of esophageal cancer. Esophageal squamous cell carcinoma (ESCC) predominates in certain parts of Asia, such as China and Japan, whereas esophageal adenocarcinoma (EAC) is prevalent in European and North American countries.^26,27^ EAC typically evolves based on the metaplasia-dysplasia-carcinoma sequence due to chronic gastroesophageal reflux disease and resultant Barrett’s metaplasia. The incidence of early-onset EAC has been increasing in the U.S.^28^ Studies have shown differences in clinical and molecular features between early-onset and later-onset EAC (Table 1).^29,30^

**Table 1.** Clinical, pathological, and tumor molecular features of early-onset gastrointestinal tract cancers.

Cancer type	Clinical and pathological features^(references)^	Molecular features^(references)^
Esophageal adenocarcinoma	Advanced disease stage at diagnosis^29,30^ High body mass index^30^ Better OS in EAC from the SEER program and the National Cancer Database in the United States^29^	SNPs in apoptotic genes (*NOS3, BCL2, CASP8*, and *TNFRSF10A*)^30^
Gastric cancer	Women and Asian/Pacific Islander^38^ Advanced disease stage at diagnosis^35^ Family history of other neoplasms^41^ Higher grade, signet-ring cell, or diffuse-type histology^35,41^	More mutations in *RHOA, ARID1A, MUC5B, BANP, CDH1, TGFBR1, TP53*^42,44^ Hypermethylation of the promoter in *EIF4E*^45^ EBV or genomically stable subtype^35^
Colorectal cancer	Family history of colorectal cancer in a first-degree relative, advanced disease stage at diagnosis, distal colon and rectal cancer, longer time to diagnosis^50,52^ Poor differentiation, lymphovascular invasion, signet-ring cell morphology, lower degree of antitumor immune response^50,55,57,68^ More aggressive features with lymph node metastasis, synchronous metastatic presentation, extrahepatic disease in metastatic colorectal cancer ^53,54^ More intensive treatments, including surgical resection and intensive chemotherapy^60^ Inconsistent findings on OS^59,61,67,71^	High frequency of microsatellite instability, more mutations in *TP53* and *CTNNB1* genes in primary colorectal cancer, fewer mutation of *BRAF* gene, lower LINE-1 methylation levels in primary colorectal cancer^56,59,66,68^ More mutations in *FBXW7* and *POLE* genes in metastatic colorectal cancer^67^ A higher prevalence of MSI-high status and a lower prevalence of CIMP-high status and *BRAF* mutations in most tumor subsites along cecum to rectum.^69^ Upregulated expression of microRNAs (*MIR4304, MIR513A, MIR628, MIR 194, MIR193A, MIR210, MIR4453*)^70^

CIMP, CpG island methylator phenotype; EAC, esophageal adenocarcinoma; EBV, Epstein-Barr virus; LINE-1, long interspersed nucleotide element-1; MSI, microsatellite instability; OS, overall survival; SEER, Surveillance, Epidemiology, and End Results; SNP, single-nucleotide polymorphisms.

Using data from the Surveillance, Epidemiology, and End Results (SEER) program and the National Cancer Database (NCDB) in the U.S., Kolb et al. demonstrated clinical features and survival outcomes of early-onset EAC.^29^ Early-onset EAC was associated with stage IV disease.^29^ However, median overall survival was longest for early-onset EAC (15.2 months) followed by middle age EAC (age 50 to 69 years), and was shortest for old age EAC (age ≥70 years, 10.4 months) (*P* <.001).^29^ In multivariate analyses, early-onset EAC was associated with better overall survival (hazard ratio [HR], 0.94; 95% confidence interval [CI], 0.92–0.96) compared to later-onset EAC.^29^

The increasing incidence of EAC in developed countries may be attributable to the westernized diets and lifestyle. However, the issue of early-onset esophageal cancer has also been recognized in developing countries.^55^ A study of ESCC patients aged 30–44 years vs. ≥45 years in Tanzania identified risk factors specifically for the younger group, including infrequent teeth cleaning, secondhand smoking, and pest infestation of grains and nuts. Of note, all these factors may affect the microbial composition of the host.^56^

### Gastric cancer

Gastric cancer is the fifth most common and one of the most deadly cancers worldwide.^57^ Adenocarcinoma (including signet-ring cell carcinoma) is the most common histopathologic type, while less common types include gastrointestinal stromal tumor, MALT (standing for mucosa-associated lymphoid tissue) lymphoma, etc.^58^ In this section, gastric cancer in most studies refers to adenocarcinoma; however, histological types may not be clearly determined in some studies. Gastric adenocarcinomas can be also divided into tumors in the cardia (the upper part of the stomach adjoining the esophagus) and non-cardia (the middle and distal part of the stomach including the fundus, body, antrum, and pylorus) according to anatomic subsite. An increasing incidence of adenocarcinoma in the gastric cardia has been observed in North America and Western Europe. Gastric non-cardia cancer has been common in Eastern and Central Asia and Eastern Europe.^58^ The incidence of gastric adenocarcinoma has steadily declined worldwide over the past 50 years due to the increasing availability of the eradication therapy of *Helicobacter pylori*.^57^ Paradoxically, the incidence of early-onset gastric cancer has increased in Western countries.^32,59,60^ Studies have shown differences in clinical and molecular features between early-onset and later-onset gastric cancers (Table 1).^31–37,61,62^

According to data from the SEER program and the NCDB in the U.S., early-onset gastric cancer is associated with Asian/Pacific Islander ancestry, poorly differentiated histologic grade, signet-ring morphology, Lauren diffuse-type histology, and stage IV disease at diagnosis.^31,32^ Data from Eastern Asia have shown an association between a family history of gastric cancer and early-onset gastric cancer.^33,61,62^

Using data from next-generation sequencing of tumors in early-onset gastric cancer patients, Setia et al. have shown a higher rate of *CDH1* mutations (22% vs. 11%; *P* =.004) but a similar rate of *TP53* mutations in early-onset gastric carcinomas compared to later-onset carcinomas.^34^ Studies of genomic analyses in early-onset diffuse-type gastric carcinomas have shown significant mutated genes, such as *BANP*, *MUC5B*, *RHOA*, *ARID1A*, and *TGFBR1*.^35,36^ Using clinical and genomic data from the Cancer Genome Atlas (TCGA), Bergquist et al. found that the early-onset gastric cancer was associated with Epstein-Barr virus infection and a genomically stable subtype.^32^ Ge et al. performed comparative genome-wide analysis of DNA methylation between early-onset and later-onset gastric cancers, and found that the hypermethylation of *EIF4E* promoter was associated with early-onset gastric cancer.^37^ These findings suggest that early-onset gastric cancers show different genomic and epigenomic alterations from later-onset gastric cancers.

### Colorectal cancer

Colorectal cancer is the second most deadly cancer and third most common malignancy worldwide.^63^ By far the most common histopathological type is adenocarcinoma (including mucinous and signet-ring cell carcinomas).^64^ The incidence of colorectal cancer has increased in patients younger than 50 years of age in high-income countries across North America, Europe, and Oceania where colorectal cancer incidence in older adults has been stable or declining.^65,66^ Accumulating evidence indicates differences in clinical and molecular features between early-onset and later-onset colorectal cancer (Table 1).^38–50,52–54,67–71^

Studies have shown that early-onset colorectal cancer may be associated with a family history of colorectal cancer, advanced disease stage (i.e., stage III or IV) at diagnosis, distal colon and rectal localization, and longer time to diagnosis compared to later-onset colorectal cancer.^38–40,45,46^ Early-onset colorectal cancer has been pathologically associated with poor differentiation, signet-ring cell morphology, lymphovascular invasion, and a lower degree of antitumor immune response.^38,41–43^

Studies have shown inconsistent data on prognosis in early-onset vs. later-onset colorectal cancer patients. Using data on patients with stage II or III colon cancer from 6 clinical trials of adjuvant chemotherapy, Fontana et al. showed that, compared to later-onset patients, early-onset colon cancer patients were more likely to experience recurrence and cancer-specific mortality (despite receiving a higher adjuvant treatment intensity).^67^ In pooled data on 35,713 patients with stage III colon cancer from 25 clinical trials, no significant differences in overall or disease-free survival were observed between early-onset and later-onset patients in multivariable analyses adjusting for tumor molecular features including mismatch repair deficiency, and *KRAS* and *BRAF* mutations.^48^ In metastatic colorectal cancer, there was no statistically significant difference in overall survival between early-onset and later-onset patients although early-onset patients were more likely to receive surgical resection or intensive chemotherapy.^47,49^ In a cohort study of 769,871 cases with primary colorectal cancer from the NCDB in the U.S., early-onset colorectal cancer was associated with better overall survival compared with later-onset disease after adjusting for disease stage.^51^

Colorectal cancers consist of heterogeneous tumors with differences in genomic and epigenomic alterations, and the tumor microenvironment through interactions between exposures including diets and lifestyle, microorganisms, and immune cells.^72,73^ The prevalence of high-level microsatellite instability (MSI) in early-onset colorectal cancer is reported to be 8% to 26%.^68–71^ Lieu et al. performed next-generation sequencing of 18,218 colorectal cancer specimens and compared genomic landscapes between early-onset and late-onset colorectal cancers.^52^ In non-MSI colorectal cancers, *TP53* and *CTNNB1* mutations were associated with early-onset colorectal cancer.^52^ In MSI-high colorectal tumors, early-onset colorectal cancer was more likely to harbor mutations in *APC* and *KRAS* and less likely to harbor mutations in *BRAF* compared to later-onset cancer.^52^ In pooled data from 2 clinical trials of metastatic colorectal cancer, *FBXW7* and *POLE* mutations were associated with early-onset disease.^50^

Several studies demonstrate epigenomic alterations in early-onset colorectal cancer. Akimoto et al. examined methylation levels of long interspersed nucleotide element-1 (LINE-1), which has been well correlated with the global DNA methylation status,^74–77^ and found that early-onset colorectal cancer was associated with LINE-1 hypomethylation.^44^ A consortium study of 14,004 cases with colorectal cancer, including 3,089 early-onset cases, found that the proportions of MSI-high, CpG island methylator phenotype (CIMP)-high, *BRAF* mutated early-onset colorectal cancers gradually increased from the rectum (8.8%, 3.4%, and 3.5%, respectively) to ascending colon (46% MSI-high; 15% CIMP-high) or transverse colon (8.6% *BRAF* mutated) (all *P*_trend_ <.001 across the rectum to ascending colon), and that later-onset MSI-high colorectal cancers showed a continuous decrease in *KRAS* mutation prevalence from the rectum (36%) to ascending colon (9%; *P*_trend_ <.001), followed by an increase in the cecum (14%).^53^ These gradual changes in molecular features of early-onset and later-onset MSI-high colorectal cancers suggest the potential role of the microbiome in the pathogenesis of both early-onset and later-onset colorectal cancer. Overall evidence suggests that differential exposure to the microbiota and their metabolites across different colorectal subsites may lead to differential tumor-microbe-immune interactions and varying developmental processes of colorectal tumor molecular subtypes along the colorectal length in various age groups. Nakamura et al. found that 7 microRNAs (*MIR4304*, *MIR513A*, *MIR628*, *MIR194*, *MIR193A*, *MIR210*, and *MIR4453*) were significantly upregulated in early-onset colorectal carcinomas compared with late-onset carcinomas, and that 4 of the 7 microRNAs (*MIR513A*, *MIR628*, *MIR193A*, and *MIR210*) were detectable in plasma specimens from the early-onset colorectal cancer patients.^54^

### Hepatocellular carcinoma (HCC)

Primary liver cancer remains a global health problem and its incidence is growing worldwide. The number of new cases of liver cancer is predicted to increase by 55% in the next two decades, with 1.4 million new diagnoses forecasted for 2040.^78^

HCC, the most common type of primary liver cancer, is the sixth most common cancer and the third leading cause of cancer-related mortality.^79^ Although there is limited data published on early-onset HCC, several studies suggest differences in clinical and molecular features between early-onset and later-onset HCC (Table 2).^80–82^

**Table 2.** Clinical, pathological, and tumor molecular features of early-onset hepatobiliary-pancreatic cancers.

Cancer type	Clinical and pathological features^(references)^	Pathological and molecular features^(references)^
Hepatocellular carcinoma	Family history of HCC or HBV infection^80,81^	Germline mutations in *SPRTN* gene^82^
Biliary tract cancer	Advanced disease stage at diagnosis, extrahepatic disease, poorly differentiated histology, and worse OS in stage IV disease in cholangiocarcinoma ^90^	Mutations in the *ASXL1* and *KMT2C* genes in cholangiocarcinoma^90^ SNPs in the *PARP1* gene in gallbladder carcinoma^91^
Pancreatic cancer	Family history of pancreatic cancer in a first-degree relative, smoking, alcohol consumption, obesity, diabetes, advanced disease stage at diagnosis^95,97,98^ Poor differentiation and perineural invasion^99,100^ Inconsistent findings on OS^95,97,99,104^	Germline mutations in *BRCA1, BRCA2*, and mismatch-repair genes^105^ SNP at 13q22.3^106^ Mutations in the *SMAD4* gene^104^

HBV, hepatitis B virus; HCC, hepatocellular carcinoma; OS, overall survival; SNP, single-nucleotide polymorphisms.

Observational studies have shown that a family history of HCC or hepatitis B virus (HBV) infection is associated with early-onset HCC.^80,81^ Although further studies are needed, nonalcoholic fatty liver disease and nonalcoholic steatohepatitis have substantially risen in prevalence among adolescents and young adults,^93^ which may lead to further rise of early-onset HCC in the future. Lessel et al. reported germline mutations in *SPRTN* among 3 patients from 2 unrelated families presenting early-onset HCC, and demonstrated that *SPRTN* dysfunction leads to sustained DNA replication stress and consequent replication-related DNA damage *in vitro* and *in vivo*.^82^

### Biliary tract cancer

Biliary tract cancer is a relatively rare malignancy arising from epithelial cells in the intrahepatic or extrahepatic bile ducts, or gallbladder.^94^ Primary sclerosing cholangitis, inflammatory bowel diseases, gallstones, liver fluke infections, biliary malformations, hepatolithiasis, hepatitis C, and liver cirrhosis have been associated with increased incidence of biliary tract cancer.^95^ Gallbladder carcinoma is the most common cancer of the biliary tract, characterized by a very poor prognosis when diagnosed at advanced stages owing to its aggressive behavior and limited therapeutic options.^96^ Studies from the U.S., Europe, and Japan have identified an increase in the incidence of biliary tract cancers in younger adults.^97–99^ Several studies have studied molecular features of early-onset biliary tract cancer (Table 2).^83,84^

A study of clinical and genomic features of cholangiocarcinoma in adolescents and younger adults by Feng et al. has shown that compared to older patients (>45 years old), younger patients with cholangiocarcinoma (≤45 years old) were more likely to have extrahepatic cholangiocarcinoma (29% vs. 17%), poorly differentiated histology, stage III or IV disease, and mutations in the *ASXL1* (11% vs. 1%) and *KMT2C* (19% vs. 4.7%) genes.^83^ In patients with stage IV cholangiocarcinomas who underwent systemic chemotherapy, the younger group was associated with worse overall survival than the older group (HR, 3.01; 95% CI, 1.14–4.91; *P* = .03).^83^ A study of gallbladder cancer by Kumari et al. suggested that a minor allele G and homozygous genotype GG of *PARP1* rs1136410 (A/G) are significantly associated with the development of gallbladder carcinoma in younger adults.^84^

### Pancreatic cancer

Pancreatic cancer is the 12th most common cancer worldwide,^2^ and the fourth leading cause of cancer-related death in both men and women in the U.S.^100^ Pancreatic cancer has a 5-year survival rate of 6% to 10% and is projected to become the second leading cause of cancer-related mortality in the U.S. by 2040.^101^ Although early-onset pancreatic cancer accounts for only 5% to 12% of all pancreatic cancer cases, an increase in the incidence of early-onset pancreatic cancer has occurred in the U.S. and other high-income countries over the past few decades.^7,8,102^ Accumulating evidence indicates differences in clinical and molecular features between early-onset and later-onset pancreatic cancer (Table 2).^85–89,103,104^

Epidemiological studies have shown that potential risk factors for early-onset pancreatic cancer include a family history of pancreatic cancer, smoking, alcohol consumption, obesity, and diabetes.^85–87,103^ A case-control study in the U.S. reported that individuals who were overweight or obese at the ages of 20 to 49 years were associated with an earlier onset of pancreatic cancer by 2 to 6 years compared to individuals with normal weight.^105^ Pathological features of early-onset pancreatic cancer have been reported to be poorly differentiated histology and perineural invasion.^88,89^

Inconsistent data on prognosis of patients with early-onset pancreatic cancer have been reported. Using data on 72,906 patients with pancreatic ductal adenocarcinoma from the SEER database in the U.S., Ansari et al. have shown that early-onset pancreatic ductal adenocarcinoma is associated with worse 5-year overall survival among all patients (6.1% vs. 8.6%; *P* = .003) and operated patients (17.7% vs. 26.9%; *P* < .001) compared to those with later-onset disease.^86^ Another study using data on 248,634 patients with pancreatic cancer from the SEER database has shown that early-onset pancreatic ductal adenocarcinoma is associated with better overall survival compared to those with later-onset disease across all disease stages.^104^ Some single-institution studies have shown no significant survival difference between early-onset and later-onset pancreatic cancers.^85,87,88^

The development and progression of pancreatic cancer are affected by somatic mutations and epigenomic regulations in neoplastic cells, and interactions between neoplastic cells, immune cells, and the microbiome in the tumor microenvironment.^106^ Ben-Aharon et al. performed next-generation sequencing of 293 pancreatic ductal adenocarcinoma specimens and found that mutations in *SMAD4* were significantly more prevalent in early-onset pancreatic cancer than in late-onset cancer.^90^ Bannon et al. examined germline DNA sequencing on pancreatic cancer susceptibility genes and found that germline mutations in *BRCA1*, *BRCA2*, or mismatch-repair genes were significantly more prevalent in early-onset pancreatic cancer compared with later-onset disease.^91^ A genome-wide association study by Campa et al. suggested a SNP at 13q22.3 as a risk allele for early-onset pancreatic cancer.^92^

## Microbiome and digestive system tumor development

### Esophageal cancer

Dysbiosis of the oral microbiota^107–109^ and *Fusobacterium nucleatum* (now termed *Fusobacterium animalis*) in tumor tissues^110^ have been associated with ESCC or EAC. These findings have been supported by the recent study by Nomburg et al., which have reported the oral and tumor microbiome from 299 patients with ESCC in five different countries (Tanzania, Malawi, Kenya, China, and Iran) with high incidence of ESCC.^111^ They found that cancer-associated, traditionally oral bacteria including the genera *Fusobacterium*, *Selenomonas*, *Prevotella*, *Streptococcus*, *Porphyromonas, Veillonella*, and *Campylobacter*, were highly enriched in ESCC tissues. In addition, they also demonstrated that there was a significant correlation between the compositions of the saliva microbiome and ESCC tumor microbiome.^111^ Hao et al. have reported that the tissue-adherent microbiome in the mouth, esophagus, stomach, and rectum from 37 patients with gastroesophageal reflux, 32 with Barrett’s esophagus, 25 with EAC, and 27 healthy individuals.^112^ They found that *Burkholderia*, *Lautropia*, *Ralstonia*, and *Veillonella* in the esophagus positively correlated with disease progression along a spectrum from gastroesophageal reflux to Barrett’s esophagus to EAC.^112^ These clinical studies suggest dysbiosis of the oral or gut microbiome may influence the development of ESCC and EAC, although exact mechanisms underlying influences of the microbiome on esophageal carcinogenesis remain to be elucidated.

### Gastric cancer

Metagenomic analyses of gastric mucosal microbiota showed that a lower microbial diversity, a lower amount of *Helicobacter*, and higher amounts of certain members of the oral microbiota, including *Parvimonas micra*, *Peptostreptococcus stomatis*, and *Fusobacterium nucleatum*, were observed in both cardia and non-cardia gastric cancer tissues, compared with nontumor tissues.^113,114^ A recent study has shown that the dysbiosis of the gastric microbiome is associated with the development of gastric cancer after *Helicobacter pylori* eradication.^115^ Zhou et al. have examined the fecal and tumor microbiome using 16S ribosomal RNA gene analysis from 1,043 gastric neoplasia patients in China, and found significant enrichments of *Streptococcus anginosus* and *Streptococcus constellatus* in gastric carcinoma tissue and stool specimens from patients with intraepithelial neoplasia, early and advanced gastric carcinoma.^116^ Emerging evidence indicates that dysbiosis of the fungal microbiome (mycobiome) is associated with human digestive system cancers.^117^ *Candida* species have been detected in gastric adenocarcinoma tissues and high abundance of *Candida* species has been associated with early-stage gastric adenocarcinoma.^117^ The presence of *Candida* species was associated with the expression of genes involved in cytosolic DNA sensing, Toll-like receptor signaling, and Nod-like receptor signaling in gastric adenocarcinomas.^117^ These findings suggest potential roles of the oral and gastric microbiome inclusive of mycobiome during gastric carcinogenesis.

### Colorectal cancer

Accumulating evidence suggests pathogenic roles of colorectal cancer-associated microbes, such as *Fusobacterium nucleatum* (now termed *Fusobacterium animalis*),^17,118–129^ *Peptostreptococcus anaerobius*,^130–134^ *Campylobacter jejuni*,^135^ polyketide synthase (pks) positive *Escherichia coli*,^136^ enterotoxigenic *Bacteroides fragilis*,^137–145^ *Enterococcus faecalis*,^146–149^ *Streptococcus gallolyticcus*,^150–152^ and *Clostridioides difficile*.^153^

Emerging evidence demonstrates interactions between the microbiome and host immunity. Galeano Niño et al. used spatial profiling technologies and single-cell RNA sequencing to examine the spatial distribution and localized effects of the microbiota in human colorectal cancer.^154^ They found *Fusobacterium* and *Bacteroides* to be the most dominant genera in colorectal tumors, and that bacterium-positive areas of colorectal tumors were associated with a downregulation of T cell markers, enrichment of myeloid cells, and upregulations of the immunosuppressive molecule ARG1 and the immune checkpoint protein CTLA4.^154^ Roelands et al. have reported a large multi-omic analyses of fresh-frozen specimens from 348 patients with primary colon cancer using RNA, whole-exome, deep T cell receptor, bacterial 16S rRNA gene, and whole-genome sequencing.^155^ They identified a tumor microbiome signature, driven by *Ruminococcus bromii*, associated with better prognosis. They also developed a composite score (mICRoScore) by the combining tumor microbiome signature and immunological constant of rejection signature, which correlates with the presence of intratumoral T helper 1 cells and cytotoxic immune responses.^155^ The mICRoScore was associated with patient survival in patients with colorectal cancer.

Several studies have explored the interplay between microorganisms and genetic and/or epigenetic alterations that promote colorectal tumorigenesis. Mouradov and colleagues performed bacterial 16S rRNA gene sequencing in tumor and normal mucosa specimens from 423 patients with stage I to IV colorectal cancer.^156^ They identified three oncomicrobial community subtypes (OCSs) with distinguishing features. OCS1 (*Fusobacterium*/oral pathogens, proteolytic, 21%) was associated with proximal location, poor differentiation, high-level MSI, high-level CIMP, consensus molecular subtype (CMS) 1, and *BRAF* and *FBXW7* mutations, whereas both OCS2 (*Firmicutes*/*Bacteroidetes*, saccharolytic, 44%) and OCS3 (*Escherichia*/*Pseudescherichia*/*Shigella*, fatty acid β-oxidation, 35%) were associated with distal location and chromosome instability.^156^ The abundance of certain microorganisms has been associated with oncogenic epigenetic alterations and upregulations of specific non-coding RNAs in colorectal cancer.^157^ *Fusobacterium nucleatum* may influence tumor overexpression of *MIR21* and *MIR1322*, which has been shown to potentiate the development and progression of colorectal tumors in mouse models.^134,158^ DeStefano Shields et al. have shown that colonization by enterotoxigenic *Bacteroides fragilis* in *Apc*/*Braf*-mutant mice developed tumors, which were infiltrated by CD8 T cells and sensitive to anti-CD274 (PD-L1) treatment.^159^ These findings suggest interactive influences of gut microorganisms and host genetic and epigenetic alterations.

High-throughput sequencing analyses have detected fungi and viruses in colorectal mucosal tissue and fecal specimens, and revealed that dysbiosis of the viral microbiome (virome) or the fungal microbiome (mycobiome) is associated with colorectal cancer.^160,161^ Some viral taxa, such as *Orthobunyavirus*, *Inovirus*, or *Tunalikevirus*, have been enriched in fecal specimens from patients with colorectal cancer, and dysbiosis of the enteric virome has been associated with worse clinical outcomes in colorectal cancer.^160^ The role of the gut fungal microbiota in the etiology and progression of colorectal cancer remains uncertain due to the low abundance of fungi in the human gut (≤0.1%–1% of total microorganisms) as well as lack of well-characterized reference genomes for aligning sequencing reads. Coker et al. found that an altered fecal mycobiome composition was associated with colorectal cancer, and that fecal specimens from colorectal cancer patients had higher amounts of *Malasseziomycetes* and lower amounts of *Saccharomycetes* and *Pneumocystidomycetes* compared to cancer-free individuals.^161^ Lin and colleagues have performed meta-analyses to pool studies with shotgun metagenomic sequencing data from 1,329 individuals, and revealed that several fungal species, including *Aspergillus rambellii*, *Cordyceps* sp. RAO-2017, *Erysiphe pulchra*, *Moniliophthora perniciosa*, *Sphaerulina musiva*, and *Phytophthora capsica*, are enriched and *Aspergillus kawachii* is depleted in fecal specimens from colorectal cancer patients.^162^ Multi-kingdom analyses of the presence of bacteria, fungi, archaea, and viruses in fecal specimens from colorecta cancer patients have revealed transkingdom (fungi-bacteria) interactions.^162,163^ Fecal specimens enriched with *Aspergillus rambellii*, also harbor high amounts of *Fusobacterium* and *P*. *micra* in patients with colorectal cancer, suggesting the transkingdom interactions between enteric fungi and bacteria during colorectal tumor progression,^162^ although the underlying mechanisms for this interactions between certain fungi and bacteria in colorectal carcinogenesis are unknown. Pan-cancer analyses of multiple body sites by Dohlman et al. have identified tumor-associated fungi including an enrichment of Candida in colon tumor.^117^

Accumulating evidence supports the role of microbes in mediating a pathogenic link between environmental factors, including diet and lifestyle, and colorectal carcinogenesis.^12,164^ Experimental evidence suggests that high-fat diet and cigarette smoking promote colorectal carcinogenesis through the modulation of gut microbiota and metabolites.^165^ Analyses of two longitudinal prospective cohort studies by Arima et al. have shown that the association of a Western-style diet with colorectal cancer incidence is stronger for tumors containing higher amounts of pks^+^ *Escherichia coli*.^166^ Gao et al. have identified metagenomic signatures of colorectal cancer and validated the signatures in seven published metagenomic datasets of colorectal cancer.^167^ Lee et al. have shown that there are significant differences in fecal microbial signatures between tubular adenomas and sessile serrated adenomas, and that *Flavonifractor plautii* and *Bacteroides stercoris* may influence effects of diet and medications on the development of these premalignant colon lesions.^168^ Han et al. have demonstrated that the gut commensal *Lactobacillus reuteri* can potentiate the protective effect of statins on colorectal tumorigenesis via the production of tryptophan catabolite, indole-3-lactic acid.^169^

### HCC

Accumulating evidence indicates that the gut microbiome can influence host immunity and the development of HCC.^170^ Ma et al. demonstrated that in multiple mouse models, *Clostridium* species could inhibit the accumulation of hepatic natural killer T cells, and suppress antitumor immune response against both primary and secondary liver tumors.^171^ A colonization with commensal *Clostridium* species, gram-positive bacteria involved in the conversion of primary to secondary bile acids, decreased hepatic natural killer T cells and increased liver tumor metastases. Hu et al. found that, after transplantation of the gut microbiota from healthy mice or *Lactobacillus reuteri* to carcinogen-induced primary HCC-bearing mice, the proportion of IL17A-producing group 3 innate lymphoid cells in the liver was decreased, leading to the reduced IL17A production and suppressed HCC development.^172^ Li et al. examined the intratumoral microbiome in human HBV-related HCC and identified two microbiome-based HCC subtypes, defined as bacteria-dominant and virus-dominant subtype.^173^ Higher infiltration of M2 macrophage was associated with bacteria-dominant subtype. Schneider et al. have shown that dysbiosis of the gut microbiome can recruit myeloid-derived suppressor cells and impair T-cell proliferation in the liver, and that *Akkermansia muciniphila* administration reduced myeloid-derived suppressor cells, liver injury, and liver fibrosis in mice.^174^

### Biliary tract cancer

Intrahepatic cholangiocarcinoma is the second most common primary liver cancer. Its incidence remains low in the Western world but is rising globally.^175^ Emerging evidence suggests potential interactions between the microbiome, tumor genomic alterations, host immunity, and biliary tract carcinogenesis. In a mouse model of primary sclerosing cholangitis, a decrease in gut barrier function can lead to an increased gut-derived bacteria and lipopolysaccharide in the liver and upregulation of CXCL1 expression in hepatocytes, which results in the recruitment of myeloid-derived suppressor cells in the liver and the development of cholangiocarcinoma.^176^ Dong et al. performed proteogenomic analyses of paired tumor and adjacent liver tissues from 262 patients with intrahepatic cholangiocarcinoma and identified four subgroups (S1-S4), which had diverse clinical, genomic, immunologic, and microenvironmental features; S1 showed the most abundant expression of inflammatory proteins, such as CD14, MPO, and C5AR1. S2 had the highest level of proteins related to cancer-associated fibroblasts and extracellular matrix, including FAP, POSTN, and FLT1. S3 was characterized by elevated MAPK and metabolic proteins, such as ACAT1, FASN, and IDH1. S4 retained maximum expression of adhesion and biliary-specific proteins, such as ANXA4, KRT18, and EPCAM.^177^ In addition, they have revealed significant differences in the intratumoral microbiome between these 4 subgroups. Chai and colleagues have found enrichment of *Paraburkholderia fungorum* in adjacent tissues of intrahepatic cholangiocarcinoma. *Paraburkholderia fungorum* can inhibit bile duct cancer cell migration and proliferation in *in vitro* and *in vivo* experiments.^178^

### Pancreatic cancer

Accumulating evidence indicates potential roles of the microbiome in host immunity and pancreatic tumor development. Pushalkar et al. have demonstrated that gut microbes can migrate to the pancreas and inhibit T-cell-mediated immunity against pancreatic tumors through the recruitment of myeloid-derived suppressor cells into the tumor microenvironment in a mouse model.^179^ Metagenomic analyses by Riquelme et al. found that high amounts of *Pseudoxanthomonas*, *Streptomyces*, or *Saccharopolyspora* in pancreatic cancer tissue specimens were associated with high density of CD8^+^ T cells in tumor tissues and better overall survival.^180^ Nagata et al. performed shotgun metagenomic analysis of fecal and salivary specimens from pancreatic ductal adenocarcinoma patients and cancer-free controls in Japan, Spain, and Germany.^181^ They have found significant enrichments of *Streptococcus* and *Veillonella* spp and a depletion of *Faecalibacterium prausnitzii* in the gut microbiome of pancreatic cancer patients.^181^ Ghaddar et al. have developed a computational pipeline of single-cell analysis of host-microbiome interactions in pancreatic cancer and identified a subset of pancreatic tumors harboring intracellular bacteria, which is associated with worse prognosis.^182^ Chen et al. have shown that deletion of type I collagen produced by pancreatic cancer cells can inhibit pancreatic tumor progression, enhance T cell infiltration, change tumor microbiome with increased *Camplyobacterales*, and potentiate efficacy of anti-PDCD1 (PD-1) immunotherapy in mice.^183^ Analyses by Aykut et al. revealed that *Malassezia* was enriched in human pancreatic cancer tissue specimens, and that *Malassezia* could potentiate the development of pancreatic tumors in a mouse model.^184^ Aftab et al. demonstrated that intratumoral mycobiome enhanced cancer cell secretion of pro-inflammatory cytokine IL33 as a chemoattractant for CD4^+^ type 2 helper T cells cells and group 2 innate lymphoid cells in the pancreatic tumor microenvironment.^185^ Depletion of fungi or deletion of IL33 in cancer cells was shown to significantly inhibit the progression of pancreatic ductal adenocarcinoma.^185^

### Current evidence for links between the microbiome and early-onset digestive system cancers

Potential risk (or protective) factors for early-onset digestive system cancers include lifestyle and environmental exposures, including obesity, physical inactivity, diet, antibiotics, and aspirin and non-steroidal anti-inflammatory drugs (NSAIDs), all of which have been shown to alter the human microbiome.^19,25,122,123,164,186–188^ Although there are limited data published on the relationship between the microbiome and early-onset digestive system cancers, studies have suggested differences in the microbiome of stool specimens or tumor tissue between early-onset and later-onset gastrointestinal cancers (Table 3).^189–191,193^

**Table 3.** Associations between the microbiome and early-onset gastrointestinal cancers.

Cancer type	Findings^(Reference)^
Gastric cancer	The amounts of *Streptococcus*, *Veillonella*, *Tyzzerella*, and *Aggregatibacter* were increased, while the amounts of *Odoribacter* and *Eubacterium ventriosum* were decreased in fecal specimens from patients with early-onset gastric cancer compared to age-matched healthy individuals.^189^
Colorectal cancer	Diversity and composition of gut microbiome are significantly different between patients with early-onset and late-onset colorectal cancer. Some specific bacterial genera, such as *Veillonella*, *Odoribacter*, *Flavonifractor*, *Actinomyces*, *Porphyromonas*, or *Fusobacterium*, were enriched in fecal specimens from patients with early-onset colorectal cancer compared to age-matched healthy individuals.^190^ The enrichment of *Bifidobacterium* genus in colorectal cancer tissue has been associated with signet-ring cell morphology that has been associated with early-onset colorectal cancer.^191^ Amounts of red meat intake-related microbes as well as *Flavonifractor plautii* were higher in fecal specimens from early-onset colorectal cancer patients than those from age-matched healthy individuals.^192^
Hepatocellular carcinoma	A viral exposure signature based on a high-throughput sequencing method was associated with the risk of developing early-onset hepatocellular carcinoma.^193^

**Figure 2.**
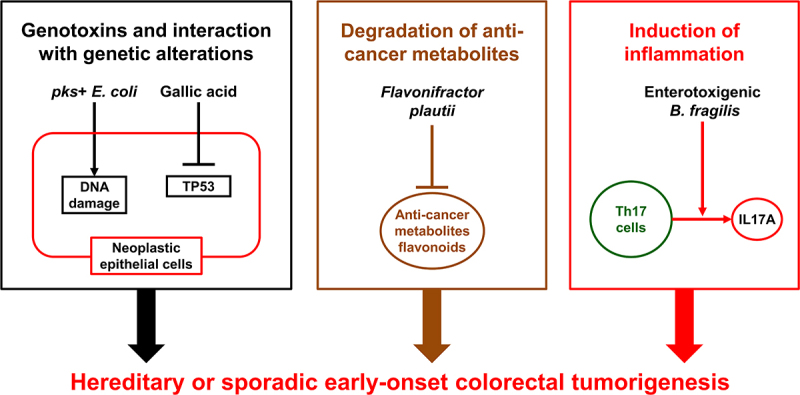
Potential interactions of the gut microbiome with host genetics, metabolism, and immunity during the development of early-onset colorectal cancer. The gut microbiota-derived gallic acid can inhibit the tumor-suppressive function of mutant TP53, which is found more frequently in early-onset colorectal cancer than in later-onset colorectal cancer. *pks*^+^ *Escherichia coli* and enterotoxigenic *Bacteroides fragilis* can increase DNA damage in colonic epithelium and induce IL17A and colonic inflammation. *Flavonifractor plautii* can degrade flavonoids, which have been shown to possess a wide variety of anti-cancer effects by modulating reactive oxygen species-scavenging enzyme activities, arresting the cell cycle, inducing apoptosis and autophagy, and suppressing cancer cell proliferation and invasiveness.

Kadosh et al. have demonstrated a relationship between the gut microbiome and host genetics during colorectal carcinogenesis. They have shown that the gut microbiota-derived gallic acid can inhibit the tumor-suppressive function of mutant TP53 and can promote the development of distal colon tumors in mice harboring the *Tp53* p.R270H mutation via hyperactivation of WNT signaling.^194^ The prevalence of *TP53* and *CTNNB1* mutations has been shown to be higher in early-onset colorectal cancer than in later-onset colorectal cancer.^52^ Familial adenomatous polyposis is caused by germline *APC* mutations.^195,196^ If left untreated, approximately half of the individuals with familial adenomatous polyposis have been shown to diagnose with colorectal cancer at an age younger than 50 years.^71^ Dejea et al. have identified that *pks*-positive *Escherichia coli* and enterotoxigenic *Bacteroides fragilis* were more commonly found in colorectal tissues from patients with familial adenomatous polyposis (68% and 60%, respectively), compared to those from healthy individuals (22% and 30%, respectively). In mouse models, co-colonization with these two microbes can potentiate intestinal carcinogenesis through increased DNA damage in colonic epithelium and IL17 induction in the colon.^197^ Metagenomic analyses of 1,038 fecal specimens from patients with colorectal cancer and age-matched healthy individuals have demonstrated that diversity and composition of the gut microbiome were significantly different between early-onset and later-onset colorectal cancers, and that amounts of some specific bacterial genera, such as *Veillonella*, *Odoribacter*, *Flavonifractor*, *Actinomyces, Porphyromonas*, and *Fusobacterium*, were higher in fecal specimens from early-onset colorectal cancer patients than those from age-matched healthy individuals.^190^ Especially, bacterial species of *Flavonifractor plautii* were enriched in fecal specimens from early-onset colorectal cancer patients. Another study of integrated metagenomic and metabolomic analyses of fecal specimens from patients with colorectal cancer by Kong et al. have demonstrated that amounts of *Flavonifractor plautii* were higher in fecal specimens from early-onset colorectal cancer patients than those from age-matched healthy individuals.^198^ *Flavonifractor plautii* has been shown to be enriched in stools from Indian patients with colorectal cancer.^199^ *Flavonifractor plautii* can degrade flavonoids that have been shown to possess a wide variety of anti-cancer effects by modulating reactive oxygen species-scavenging enzyme activities, arresting the cell cycle, inducing apoptosis and autophagy, and suppressing cancer cell proliferation and invasiveness.^200,201^ These findings suggest that *Flavonifractor plautii* may potentiate the development of early-onset colorectal cancer through the degradation of flavonoids, underscoring potential influence of the gut microbiome on host genetics, metabolism, and immunity during tumor development (Figure 2).

The enrichment of *Bifidobacterium* genus in colorectal cancer tissue has been associated with signet-ring cell morphology,^191^ which has been a pathological feature of early-onset colorectal cancer.^42,202^ *Bifidobacteria* can modulate intestinal epithelial cell differentiation factors in *in vitro* and *in vivo* experiments.^203^ *Bifidobacterium* genus might influence early-onset colorectal tumorigenesis by modulating epithelial cell differentiation, although further studies are needed to clarify exact mechanisms.

Comparative studies of the gut microbiome in digestive system cancers have found significant differences in the gut microbiome or blood virome between early-onset gastric cancer or HCC and age-matched healthy individuals. Chen et al. have performed 16S rRNA gene sequencing of fecal specimens from 196 patients with gastric cancer and 30 healthy individuals in China, and showed that the amounts of *Streptococcus*, *Veillonella*, *Tyzzerella*, and *Aggregatibacter* were increased, while those of *Odoribacter* and *Eubacterium ventriosum* were lower in stools of early-onset gastric cancer patients than those of age-matched healthy individuals.^189^ Virome analyses by Liu et al. have demonstrated a blood-based viral exposure signature to predict the occurrence of HCC, and suggested pathogenic roles of viruses other than hepatitis B virus and hepatitis C virus in the development of HCC.^193^ Further studies are needed to clarify exact mechanisms underlying these associations of the microbiome with early-onset digestive system cancers.

### Challenges and emerging opportunities

There remain substantial knowledge gaps in the etiology and pathogenesis of early-onset digestive system cancers. It is speculated that dietary, lifestyle, and environmental exposures in early life (from conception to adolescence) may be potential risk factors for the early-onset digestive system neoplasms.^3,5,6,204^ However, comprehensive analyses of risk factors in early life and young adulthood are difficult without proper study design and data collection. Compared to the prospective design of data and biospecimen collections, it would be much easier to design a case-control study with data and biospecimen collections from cases as well as controls. However, many pitfalls such as differential recall bias and reserve causation exist.^25^ In the context of microbiome research, reverse causation imply that the presence of disease such as a gastrointestinal disease in cases may influence measured analytes in biospecimens such as stool.^25^ Utilizing data from existing cohort studies that enrolled early-life participants combined with collections of biospecimens (including blood, stool, saliva, urine, tissue, etc.) enables us to examine early-life exposures in relation to the development of early-onset digestive system cancers.^205–207^ The conceptualization of combining the molecular pathological epidemiology (MPE) analytical framework into lifecourse epidemiology designs to assess pathogenic effects of early-life factors on diseases later in life has also been developed.^208^

Reproducibility is another challenge in microbiome research. It has been shown that findings from different studies may be quite disparate. This appears to be due to multiple factors including complex microbial communities, preanalytical and analytical factors, measurement errors, chance variation, among others. Internal and external validations are essential in any given study. It is important to develop rigorous, standardized approaches in microbiome research.

Utilizing the microbiology-MPE research, we can investigate exposures, including diets, lifestyle, and the microbiome in relation to specific cancer subtypes defined by tissue microbial profiling.^24^ A previous microbiology-MPE study has shown that a so-called prudent diet rich in whole grains and dietary fiber is associated with low incidence of colorectal carcinoma having tissue *Fusobacterium nucleatum* but not carcinoma without *F*. *nucleatum*.^188^ A further study has revealed a link between inflammatory diets and colorectal carcinomas with *Fusobacterium nucleatum*, especially in the proximal colon, suggesting that the intestinal inflammatory processes may facilitate colorectal carcinogenesis via altering mucosal barrier functions or augmenting tumorigenic effects of *Fusobacterium nucleatum*.^209^ Experimental and observational studies have shown that *Fusobacterium nucleatum* may contribute carcinogenesis via promoting proliferations of neoplastic clones or suggesting anti-tumor immune reactions.^122,123,131,133,210,211^ Another putative pathogenic bacterium for colorectal carcinomas is colibactin-producing *pks*+ *Escherichia coli*. Colibactin has been shown to cause a specific pattern of genomic mutational changes in colorectal epithelial cells.^212^ A western-style diet rich in red and processed meat, sugar, and refined grains is another major dietary patterns in the U.S. A microbiology-MPE study has shown that the western dietary pattern is associated with increased incidence of colorectal carcinoma with high-level tissue *pks*^+^ *Escherichia coli*.^166^ These findings from microbiology-MPE studies support a potential role of bacteria in the relationship of dietary factors with colorectal carcinogenesis. If microbial data are collected from children and/or young adults in a prospective study, we can link microbial profiles with the incidence of specific early-onset cancer subtypes determined by tumor molecular characteristics (e.g. somatic mutations and epigenetic alterations) or tumor microenvironmental features (e.g. pathogenic bacteria). Another knowledge gap is a limited understanding of the pathogenic roles of the microbiome in the development and progression of early-onset digestive system tumors. Hence, further studies are needed to clarify molecular mechanisms by which the microbiome can influence the development and progression of early-onset digestive system tumors.

There is limited evidence for substantial influences of host genetics/epigenetics on the gut microbiome. Existing evidence suggests that maternal exposures, infant feeding, diets, medications, lifestyle patterns, and other environmental exposures can alter the gut microbiome in early life.^213^ Studies have shown that probiotics intake may generate antiproliferative or pro-apoptotic metabolites in the intestine,^214–216^ inhibit intestinal inflammation,^217,218^ reverse gut dysbiosis,^219,220^ and reactivate antitumor immunity,^221,222^ thereby leading to the clearance of premalignant cells^223^ and prevention of colorectal tumors. Probiotics use, which represents one of the potential prevention strategies of early-onset digestive system tumors, has their relatively safe features and potential health benefits, although further studies are required to assess its short-term and long-term impact on human health and diseases.

## Conclusion and future directions

**Figure 3.**
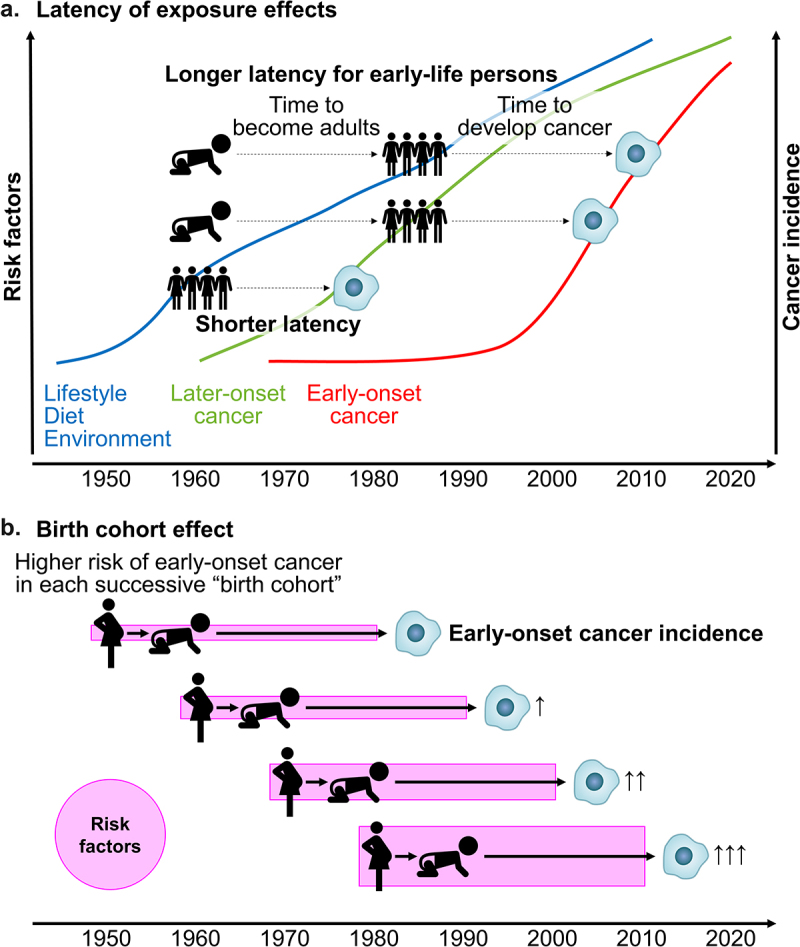
Simplified schemes illustrating factors related to the rising incidence of early-onset cancers.

The incidence of early-onset digestive system cancers, diagnosed in individuals under 50 years of age has been increasing worldwide. Although the specific causes remain largely unknown, dietary, lifestyle, and environmental changes that have been occurring across generations since the mid-20th century are considered to play etiological roles (Figure 3). The gastrointestinal microbiome is a key factor in this emerging trend. Emerging evidence suggests that the gut microbiome may influence host genetics, metabolism, and immunity during early-onset digestive system tumor development. Given potential preventative and therapeutic interventions targeting the microbiome, further research on interactive influences of environmental exposures and the microbiome on digestive system tumor development is needed. Ideally, life-course epidemiological studies combined with prospective collections of biospecimens (stool, blood, saliva, urine, placenta, cord blood, etc.) and tumor tissues will allow for microbiology-MPE research that can study pathogenesis of early-onset cancers. In the meantime, existing cohort studies can be utilized to study risk factor exposures in early life in relation to the development of early-onset cancers. Additionally, we need experimental research encompassing *in vitro* and *in vivo* studies to examine effects of the microbiota on tumor development in high-fidelity model systems as well as human intervention trial studies to assess the effects of diets, lifestyle, pre/probiotics, etc. on the intestinal microbiota and/or tumor development. Those collective research efforts will provide evidence required to design and implement intervention programs aimed at young individuals for cancer prevention. A better understanding the pathogenic influences of the gut microbiome on early-onset digestive system cancers will enable tailored screening and preventive strategies, and therapeutic interventions for these cancers.

a. Latency of exposure effects for early-onset and later-onset cancers. Note that, for simplicity, the trajectories of the levels of risk factors and the incidence of early-onset and later-onset cancers are illustrated as generalized curves, which cannot accurately represent those of individuals or populations. The y axis indicates a general scale but not absolute value. The slope of each curve does not indicate any specific increasing value, but rather shows a general rising trend only. The modernization and westernization of diets, lifestyle, and environment had started to accelerate in developed countries since the mid 20th century and in developing countries since the late 20th century.^3,224^ Due to its shorter latency of exposure effects on middle-aged or elderly adults for the development of later-onset cancers, their incidence had started to rise in around 1950s-1960s.^3,5^In contrast, exposure effects on early-life individuals did not appreciably appear until at least 3-4 decades later when those effects have been manifested as the increased incidence of early-onset cancers. Note that, after the early-life periods, young adults continue to be exposed to (the same, similar, or varying) risk factors, which may further contribute to the rise of early-onset cancers.

b. Birth cohort effect. The term “birth cohort effect” refers to an effect that appears differentially in each cohort of individuals starting at birth in/around similar time. Evidence suggests that each successive newer birth cohort have higher risk of certain early-onset cancers such as colorectal cancer.^3,5,225,226^ This phenomenon appears to be due to higher levels of risk factor exposure in early life in a given cohort compared to a preceding cohort. Studies have shown that each successive birth cohort exhibited a higher risk of developing early-onset cancer.^3,5,225,226^ This “birth cohort effect” phenomenon provides evidence for a potential etiological role of differential exposure effects during the life course (shown in Figure 1) in the recent increase in early-onset cancer.

## Abbreviations

CI: confidence interval
CIMP: CpG island methylator phenotype
CMS: consensus molecular subtype
EAC: esophageal adenocarcinoma
EBV: Epstein-Barr virus
ESCC: esophageal squamous cell carcinoma
HBV: hepatitis B virus
HCC: hepatocellular carcinoma
HGNC: HUGO Gene Nomenclature Committee
HR: hazard ratio
HUGO: Human Genome Organisation
LINE-1: long interspersed nucleotide element-1
MALT: mucosa-associated lymphoid tissue
MSI: microsatellite instability
MPE: molecular pathological epidemiology
NCDB: National Cancer Database
NSAID: non-steroidal anti-inflammatory drug
OCS: oncomicrobial community subtype
PD-1: programmed cell death 1 (PDCD1)
PD-L1: programmed cell death 1 ligand 1 (CD274)
pks: polyketide synthase
SEER: Surveillance, Epidemiology, and End Results
SNP: single-nucleotide polymorphism
TCGA: The Cancer Genome Atlas

## Use of standardized official symbols

We use HUGO (Human Genome Organization) Gene Nomenclature Committee (HGNC)-approved official symbols (or root symbols) for genes and gene products, including ACAT1, ANXA4, APC, ARG1, ARID1A, ASXL1, BANP, BRAF, BRCA1, BRCA2, C5AR1, CD4, CD8, CD14, CD274, CDH1, CTLA4, CTNNB1, CXCL1, EIF4E, EPCAM, FAP, FASN, FBXW7, FLT1, IDH1, IL17A, IL33, KMT2C, KRAS, KRT18, MAPK, MIR21, MIR193A, MIR194, MIR210, MIR513A, MIR628, MIR1322, MIR4304, MIR4453, MPO, MUC5B, PARP1, PDCD1, POLE, POSTN, RHOA, SMAD4, SPRTN, TGFBR1, TP53, WNT; all of which are described at www.genenames.org. The official gene symbols are italicized to differentiate from non-italicized gene product symbols.
